# Impact of Integrated Control Interventions on Sandfly Populations in Human and Canine Visceral Leishmaniasis Control in Araçatuba, State of São Paulo, Brazil

**DOI:** 10.3390/insects17010125

**Published:** 2026-01-21

**Authors:** Keuryn Alessandra Mira Luz-Requena, Tania Mara Tomiko Suto, Osias Rangel, Regina Célia Loverdi de Lima Stringheta, Thais Rabelo Santos-Doni, Lilian Aparecida Colebrusco Rodas, Katia Denise Saraiva Bresciani

**Affiliations:** 1 School of Veterinary Medicine, São Paulo State University (Unesp), Araçatuba 16050-680, SP, Brazil; kaml.requena@unesp.br (K.A.M.L.-R.); tania.suto@unesp.br (T.M.T.S.); loverdi.lima@unesp.br (R.C.L.d.L.S.); katia.bresciani@unesp.br (K.D.S.B.); 2 Pasteur Institute, Center for Disease Control, Sao Paulo State Department of Health, São Paulo 01027-000, SP, Brazil; orangel@pasteur.saude.sp.gov.br (O.R.); colerodas@gmail.com (L.A.C.R.); 3 Institute of Agricultural Sciences (ICA), Federal University of Jequitinhonha and Mucuri Valleys (UFVJM), Avenida Universitários, 1000, Unaí 38610-000, MG, Brazil

**Keywords:** *Leishmania*, *Lutzomyia longipalpis*, visceral leishmaniasis control

## Abstract

Visceral leishmaniasis (VL) is a serious vector-borne disease affecting humans and dogs, posing major public health challenges in endemic regions. Control efforts often target sandfly vectors, whose larvae and pupae develop in soil. Environmental management, such as removing organic matter, reducing moisture, and pruning vegetation, aims to limit breeding sites and reduce sandfly populations. This study evaluated the impact of integrated interventions on sandfly behavior in priority areas for VL control in Araçatuba, São Paulo, Brazil. The control strategy combined environmental management, canine surveys, and educational actions across seven local work areas (LWAs). Between 2019 and 2021, CDC-type light traps were installed in intra- and peridomiciliary settings at twelve properties in LWA 5. Spatial risk analysis for canine transmission was conducted in LWAs 3 and 5 using a Generalized Additive Model, with results presented as spatial odds ratios. Vector prevalence was analyzed using negative binomial regression compared to historical municipal data. Intervention coverage averaged 52.91% of visited properties (n = 15,905), ranging from 48% to 76.8% across LWAs. Adherence to environmental management exceeded 85%. Of the 150 sandflies collected, 98.67% were *Lutzomyia longipalpis* and 1.33% *Nyssomyia neivai*. A 6% reduction in vector density was observed compared with historical data, although this difference was not statistically significant. Spatial risk varied among LWAs, indicating heterogeneous transmission levels. These findings suggest that integrated environmental and educational interventions may contribute to reducing vector density and that identifying priority areas tends to support surveillance and the effectiveness of disease control actions.

## Simple Summary

Visceral leishmaniasis (VL) is a serious disease that affects humans and dogs and can be fatal if left untreated. It is transmitted by sandflies that develop in the accumulation of decomposing organic matter. To reduce the reproduction of these insects, control measures such as yard cleaning and health education activities are routinely implemented. This study evaluated how integrated actions carried out by municipal surveillance teams—including environmental management, canine blood collection to assess disease circulation, and educational activities—influenced sandfly behavior in priority areas of Araçatuba, São Paulo. Between 2019 and 2021, light traps for insect attraction were installed in households to monitor the presence of sandflies, and spatial analyses were performed to identify areas of higher risk for canine transmission. Data obtained from the municipality’s historical series were also compared with those from the study. Interventions covered more than 50% of the visited properties, and environmental management guidance exceeded 85%. A total of 150 sandflies were collected, most of them belonging to the species *Lutzomyia longipalpis*, the main vector of VL. A 6% reduction in vector density was observed compared to previous years; however, this difference was not statistically significant. Spatial analysis indicated that the risk of transmission was not uniform across the geographic area. These results suggest that integrated environmental and educational actions may contribute to reducing sandfly populations. Likewise, identifying priority areas may strengthen surveillance and improve the effectiveness of VL control measures.

## 1. Introduction

Visceral leishmaniasis (VL) is a non-contagious infectious disease with zoonotic potential, caused by *Leishmania* (*Leishmania*) *infantum* (synonym *L. chagasi*), which is transmitted by the bite of an infected female sandfly—genus *Phlebotomus* in the Old World and *Lutzomyia* in the New World [1,2,3]. The World Health Organization (WHO) estimates that between 30,000 and 90,000 new VL cases occur worldwide each year, with only 25 to 45% of cases being reported [4]. VL is endemic across many countries in the Mediterranean region, the Middle East, Asia, Central America, and South America [5], with Brazil accounting for more than 90% of cases in Latin America [4]. Dogs serve as the primary hosts and domestic reservoirs, and a high prevalence of infection in dogs significantly elevates the risk to human health [6,7,8].

The progressive spread of VL and associated socioeconomic challenges have resulted in 625 confirmed human cases and 63 deaths in the state of São Paulo, Brazil, from 2017 to 2023. During this period, the municipality of Araçatuba reported 47 cases and seven fatalities [9]. Socioeconomic factors, malnutrition, population mobility, environmental changes, and climate variability contribute to the transmission dynamics of VL. To mitigate disease spread, municipalities implement control measures targeting conditions favorable to the vector, including euthanasia of seropositive dogs, early diagnosis, and treatment of human cases. Despite these efforts, there is a rising trend in autochthonous human cases and urban endemic areas of VL in Brazil [10].

Understanding transmission patterns is essential for health authorities to prioritize interventions and optimize resource allocation. Various models and methodologies have been employed to depict distribution and predict transmission risk [11]. Monitoring and analyzing these patterns is justified given the evolving epidemiological landscape driven by ongoing human interventions [12].

Since sandfly larvae and pupae develop in the soil, their natural breeding sites are rarely visible to the naked eye, which prevents their direct elimination [13]. Thus, controlling these vectors is a challenge for surveillance programs, where environmental management is a strategic tool aimed at reducing potential breeding sites and the proliferation of sandflies [14,15]. Therefore, the strategy adopted by the Epidemiological Surveillance of the municipality of Araçatuba was to restructure its work approach through the integration of municipal surveillance services, in which Community Health Agents (ACS) from Primary Care and Endemic Disease Control Agents (ACE) work together within a single territory. Environmental management and educational actions were carried out simultaneously with the canine survey, in accordance with Federal Law No. 13,595 of 2018.

This study aimed to examine the population dynamics of sandflies in a key VL control area within the municipality of Araçatuba following integrated environmental management and educational interventions, as well as to assess the spatial risk of VL in the region.

## 2. Materials and Methods

### 2.1. Ethical Statement

Ethical approval for this study was obtained from the Research Ethics Committee (CEP) of the School of Dentistry of Araçatuba, Unesp, Araçatuba Campus, under process number 21171419.4.0000.5420.

### 2.2. Study Area

The study was conducted in Araçatuba, located in the northwestern region of the state of São Paulo, Brazil, approximately 532 km from the state capital. The city lies at a latitude of 21°12′32′′ S, longitude 50°25′58′′ W, and an altitude of 355 m above sea level. Araçatuba encompasses an area of approximately 1167 km^2^ and has an estimated population of around 207,775 inhabitants. The region experiences an average annual temperature of 23.8 °C, with minimum and maximum temperatures of 12.6 °C and 31.8 °C, respectively. The rainy season extends from October to March, with an average annual precipitation of 1267 mm [13].

Between 2019 and 2021, monitoring of VL surveillance and control activities was conducted, along with the development of risk analysis studies focusing on canine transmission, vector surveillance, and control. Operational units, known as Local Work Areas (LWAs), were defined as prioritized zones within the municipality for targeted interventions and resource allocation [14].

**Figure 1:**
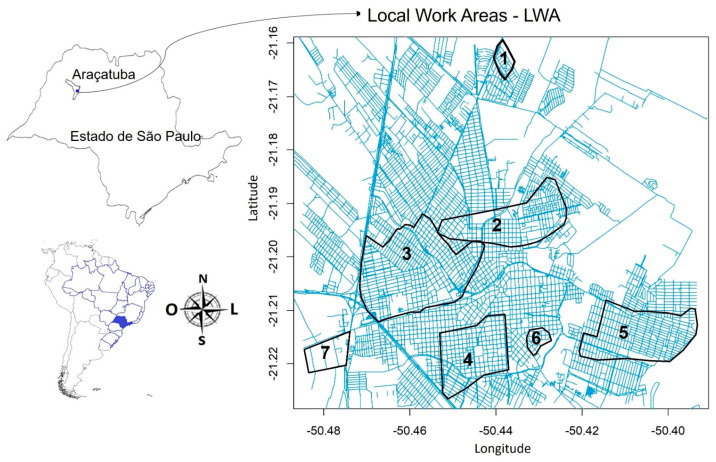
Map showing the spatial distribution of Local Work Areas (LWAs) where intervention activities were conducted in the municipality of Araçatuba, state of São Paulo, Brazil.

The definition of LWAs (Figure 1) was based on indicators including the accumulated incidence of human VL cases over the past five years, canine infection prevalence, and areas characterized by rural features or socioeconomic vulnerability. Consequently, the chronological sequence of operational focus began with LWA 5 and LWA 3. Meanwhile, LWAs 1, 7, 4, 6, and 2 were addressed selectively due to constraints imposed by the COVID-19 pandemic.

The data were consolidated in the FlebWebLV information system, managed by the Department of Health.

### 2.3. Intervention Strategy

In accordance with Federal Law No. 13,595 of 2018, the municipality of Araçatuba restructured its operational approach by integrating municipal surveillance with Community Health Agents (Agentes Comunitários de Saúde) from Basic Healthcare and Endemic Disease Control Agents (Agentes de Combate às Endemias). These teams collaborate within the same geographic territories, aligned with the network of Basic Health Units, to deepen their understanding of local contexts and design interventions tailored to community health needs. Starting in October 2019, integrated activities were conducted by pairs of Endemic Disease Control Agents, combining canine surveys, environmental management, and health education. This strategy aimed to strengthen surveillance and control efforts by identifying properties requiring urgent environmental management and maintaining monthly monitoring of these prioritized locations.

### 2.4. Educational Action

To strengthen their efforts, the Endemic Disease Control Agents received bimonthly training from June to November 2019. These sessions were facilitated by technicians from the Pasteur Institute (formerly the Superintendence of Endemic Disease Control, SUCEN), postgraduate researchers from the School of Veterinary Medicine at UNESP/Araçatuba, and staff from the Araçatuba Municipal Health Department. The training aimed to deepen the agents’ understanding of VL surveillance and control processes.

The meetings focused on enhancing the agents’ holistic perception of the environment. Through lectures and interactive sessions that incorporated visual aids and open dialogue, the agents were exposed to a wide range of topics within the VL control program. These included human leishmaniasis case notification and diagnosis, vector biology and breeding sites, canine VL surveys, responsible dog ownership and animal welfare, educational components of VL control, information systems, home visit protocols—including diagnosis and environmental management—evaluation of risk factors, practical canine survey training, and other relevant subjects.

### 2.5. Environmental Management

In parallel with the canine survey, environmental management and educational activities were fully implemented in LWAs 5 and 3, along with the evaluation of spatial risk distribution using odds ratios (OR). Investigations targeted favorable conditions for the sandfly vector, with guidance provided on eliminating organic matter, pruning trees, removing moisture sources, and managing animals, among other measures, to reduce breeding sites for immature sandfly stages. Each property underwent an environmental assessment focusing on sanitary conditions, followed by the establishment of deadlines and action plans for residents to mitigate risk factors.

### 2.6. Entomological Survey

Captures were conducted exclusively in LWA 5 from September 2019 to December 2021, with interruptions from March to December 2020 and from March to December 2021 due to the COVID-19 pandemic, resulting in a total of 12 months of active entomological investigation comprising 409 collections. First, the blocks were randomly selected. Subsequently, 12 properties were chosen by convenience sampling based on favorable conditions for the maintenance of sandflies, such as the presence of decomposing organic matter of plant origin in shaded areas and/or the presence of domestic animals (canines and/or gallinaceous birds), which serve as bloodmeal sources for sandflies.

CDC-type light traps were installed in the 12 participating households. Specifically, one trap was placed indoors and another in the peridomestic area of each property, approximately one meter above the ground. The traps remained in operation for 12 h over three consecutive nights each month, totaling 24 active traps per sampling period. One hour after sunset, residents were instructed to activate the CDC light traps, which were turned off the following morning. A technician from the Pasteur Institute replaced the collection cups each morning, and this procedure was repeated for three consecutive nights. After completion, the traps were dismantled, and the collection cups were sealed in plastic bags for transport to the medical entomology laboratory. There, specimens were immobilized by freezing, transferred to Petri dishes for separation of sandflies from other insects, and subsequently stored in entomological boxes pre-treated with a naphthalene/paraffin mixture for preservation.

Subsequently, the specimens were cleared according to the sandfly preparation technique [16]. Briefly, the specimens were treated with liquid phenol and potassium hydroxide (10% KOH) for clarification, stained with acid fuchsin, and washed through an increasing series of ethyl alcohol concentrations, after which they were immersed in eugenol. Species identification (males and females) was performed according to the classification proposed by Galati (2003) [15]. To assess the entomological captures in LWA 5, historical data from three periods were utilized: the baseline series from 2007 to 2015, data from 2016 to 2018, and data from 2019 to 2021, the latter originating from the migration of the Pasteur Institute’s SIS-ZOO database to the online platform FlebWebLV.

### 2.7. Canine Epidemiological Survey

The canine epidemiological survey was conducted by pairs of agents and involved blood sample collection followed by serological confirmation. Samples were sent to the Adolfo Lutz Institute in Araçatuba for analysis. Dogs testing positive were confirmed based on concordant positive results between the Dual Path Platform (DPP^®^) rapid test and the enzyme-linked immunosorbent assay (ELISA).

**Figure 2:**
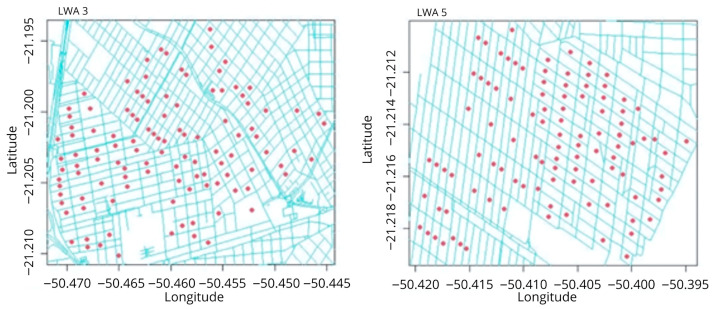
Representation of the centroids (red points) of georeferenced city blocks (cases and controls) using the Global Positioning System within Local Work Areas (LWA) 3 and 5 in the municipality of Araçatuba, state of São Paulo, Brazil.

In the two LWAs with the highest number of collections (5 and 3), a spatial case–control study was performed (Figure 2). Positive dogs were considered cases, whereas negative dogs were considered controls, and they were georeferenced at the city block level using Global Positioning System (GPS) coordinates in decimal degrees based on the WGS 84 Datum projection. Each city block was considered a probable site of infection. The analysis included only blocks that had at least one case and one control. Blocks lacking either were excluded from the study.

### 2.8. Statistical Analysis

The overall data were analyzed using percentage difference and frequency analysis, calculated according to the following equation:

$$\left(\frac{V1-V2}{V2}\right)\times 100$$

where *V*1 and *V*2 represent the values of the percentages analyzed.

The spatial case–control study was analyzed using the Generalized Additive Model (GAM) [17] from the GAM [18] library, which provided estimates of spatial odds ratios (OR). Prevalence ratio was used as a measure to compare the different series of *Lu. longipalpis* captures with those of the current study, considering an error of *p* < 0.05. Negative binomial regression II from the GAMLSS library [19] was implemented for abundance classes to estimate prevalence ratios (RPcl) [20]. The model fit was verified using the normality parameters of the residuals. The GAM and GAMLSS libraries were manipulated in the R System [21]. For analysis, GAM was used to model the risk estimated by the spatial odds ratio (OR). Prevalence ratios for abundance classes of *Lu. longipalpis* [20] were calculated using Negative Binomial Regression II, contrasting with the historical series from the municipality conducted by Pasteur Institute.

## 3. Results

The control activities carried out during the 2019–2021 triennium are summarized in Table 1, which presents the number of dogs sampled and those testing positive per LWA, the number of environmental management instructions provided per visited property, the recorded human cases, and the coverage of properties where interventions were conducted.

Of the total canine samples collected, 13.17% tested positive. The overall positivity rate varied across LWAs, ranging from 0.0% in LWA 4 to 16.13% in LWA 2. The largest number of samples was collected in LWAs 5 and 3, with LWA 5 showing a positivity rate 4.4% lower than that of LWA 3. Environmental management guidelines were 34.4% more frequently provided in LWA 5 compared to LWA 3. Except for LWA 6, all other LWAs reported between zero and four human cases, with the highest number occurring in LWA 5. In total, 13 human cases were recorded during the study period (Table 1).

LWA 2 and LWA 4 had the highest proportions of worked properties, with coverage rates of 76.8% and 67.83%, respectively, while LWA 5 had the lowest coverage at 48%. All other LWAs had over 50% of properties worked. Across all seven LWAs, the provision of environmental management guidance exceeded 85%, with LWA 5 reaching 90.6% and LWA 3 achieving 99.1%.

**Figure 3:**
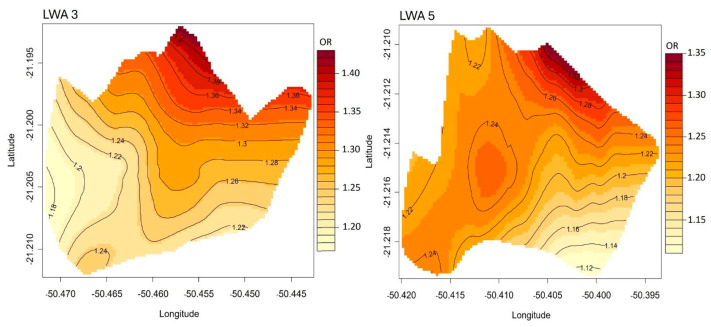
Spatial risk distribution (Odds Ratio) of visceral leishmaniasis in Local Work Areas (LWA) 3 and 5 in the municipality of Araçatuba, state of São Paulo, Brazil.

According to the spatial contour analysis illustrated in Figure 3, the distribution of spatial risk (odds ratio, OR) was heterogeneous. In both LWA 5 and LWA 3, the spatial risk of transmission varied depending on the specific area within the LWAs. The analysis indicated an increase in spatial risk toward the northern portions of these areas. In LWA 3, risk frequencies (OR) ranged from 20% to 40%, whereas in LWA 5, they ranged from 15% to 35%.

Table 2 presents a reduction in the relative population capture level (RPcl) of *Lu. longipalpis* within the study area delimited by LWA 5, compared to the baseline historical series from 2007 to 2015, the intermediate series from 2016 to 2018, and the recent series from 2019 to 2021. A 6% decrease was observed, calculated by subtracting the RPcl of the current study (0.94) from the baseline period (1.00), i.e., (1 − 0.94 = 0.06). However, this reduction was not statistically significant (RPcl = 0.9449; 95% confidence interval: 0.6587–1.3554).

During the entomological investigation in LWA 5, 150 sandfly specimens were captured, mostly in the peridomicile area (96%). Of the total, 98.67% were *Lu. longipalpis* and 1.33% were *Nyssomyia neivai*.

## 4. Discussion

Visceral leishmaniasis (VL) remains a significant public health challenge in many endemic regions, including the municipality of Araçatuba, São Paulo, Brazil. Effective control of VL requires integrated approaches that combine environmental management, vector control, reservoir management, and community education. Understanding vector dynamics and spatial risk distribution is essential for optimizing these control efforts and reducing disease transmission.

Based on the entomological investigation results, a modest 6% reduction in the population of *Lu. longipalpis* was observed in LWA 5 after integrated activities involving environmental management, educational actions, and canine surveys. However, this reduction was lower than expected when compared to the municipality’s historical data, indicating that challenges remain in sufficiently reducing vector populations.

The spatial risk of *L. infantum* transmission varied between the studied LWAs (5 and 3), with higher-risk areas correlating with observed positivity rates. Notably, an increased spatial risk was observed toward the northern part of these areas in the municipality. Identifying such priority zones is critical for focusing surveillance and control efforts to interrupt the transmission chain more effectively.

VL transmission dynamics are complex and influenced by multiple social and environmental factors. Epidemiological evidence consistently shows that canine infections often precede human VL cases, demonstrating a strong correlation between canine and human disease prevalence in urban settings [22,23]. For example, a geospatial study conducted in Presidente Prudente (SP) between 2010 and 2016 revealed VL expansion despite low canine VL prevalence [11], highlighting the nuanced relationship between reservoir presence and disease spread. On the other hand, in Campo Grande (MS), risk was found to be associated with human and canine density, as well as with environmental conditions favorable to vector proliferation [22].

Transmission patterns are heterogeneous across regions, influenced by local ecological and socioeconomic factors. This variability complicates the identification of a universal transmission model. Among analysis methods, kernel density estimation and scan statistics are widely used, but some authors recommend case–control studies employing GAMs with a binomial distribution for their relevance and robustness [24].

The Brazilian Ministry of Health recommends an integrated VL control strategy encompassing early diagnosis and treatment of human cases, vector control through insecticides, environmental management, health education, and the euthanasia of seropositive dogs [25]. The latter measure, however, remains controversial due to questions about its effectiveness and associated social, cultural, and ethical concerns [26]. Alternative approaches such as canine vaccination and the use of insecticide-impregnated collars have shown promising results [27]. The Ministry of Health has distributed pyrethroid-impregnated collars in priority municipalities, marking a significant advance in public policy despite incomplete coverage [8].

Intervention studies conducted between 2012 and 2015 demonstrated that treating over 300,000 dogs with 4% deltamethrin collars, alongside other control measures, resulted in a 50% reduction in canine VL prevalence compared to control areas. This evidence underscores the importance of combining environmental management with community-based interventions to effectively inhibit VL transmission [14].

In this study, *Lu. longipalpis* was the predominant sandfly species captured, primarily in peridomiciliary environments. This species is widely distributed across northeastern, northern, southeastern, and midwestern Brazil [16], exhibiting an eclectic feeding behavior that includes humans and other vertebrates [28]. Such ecological traits emphasize the importance of targeting peridomicile environments for vector control. Vegetation, tree trunks, and organic matter accumulation in these areas provide suitable breeding sites, reinforcing the need for environmental management strategies to disrupt vector life cycles [29].

In this study, the sampling effort totaled 864 trap-nights, corresponding to approximately 10,368 h of exposure over 12 months of collection. Entomological captures were concentrated during months with higher temperatures and rainfall (September to March), a period in which a greater population density of *Lu. longipalpis* is observed, as environmental conditions are more favorable to its development [30,31]. However, the low vector density observed may be related to several factors, including the interruption of entomological collections from March to December 2020 and during the same period in 2021 due to the COVID-19 pandemic, which affected collection frequency. Additionally, in LWA5— the priority area for HVL control—vector control measures recommended by the program were implemented [16] following the occurrence of confirmed human cases (four in 2019 and one in 2021), with insecticide application within a minimum radius of 200 m around the probable sites of human infection, in addition to environmental management and educational activities. These interventions, combined with continuous surveillance, may have contributed to reducing sandfly density.

This study presents some limitations. Entomological collections were conducted exclusively in LWA5; therefore, the findings are not generalizable. The relatively small number of sandflies captured limited the statistical power to detect more pronounced differences between the periods or areas evaluated. Nonetheless, it was possible to compare the data obtained with the historical series using prevalence ratio analysis, allowing the observation of relative increases or decreases in sandfly density compared to previous years and providing insight into the effects of the intervention measures.

In Araçatuba, coverage of properties where intervention activities were conducted fell below the expected 80%, likely influenced by the COVID-19 pandemic and resulting operational interruptions. Nevertheless, environmental management guidelines were implemented in over 85% of targeted properties. Although LWA5 had the lowest percentage of worked properties (48%), more than 90% of these received management guidance, indicating that a substantial portion of the municipality remains conducive to vector development.

Although the relative percentage reduction in vector density was not statistically significant compared to historical trends, the moderate decrease observed aligns with findings from Belo Horizonte, Minas Gerais. In that location, environmental management measures proved more effective than chemical spraying in reducing *Lu. longipalpis* populations, supporting the adoption of integrated environmental approaches for vector control. A two-year evaluation comparing chemical spraying with environmental management found that, out of 1727 sandflies collected, 267 were from areas with environmental management, 444 from chemically sprayed areas, and 1016 from untreated control areas. These results indicate that environmental management had the most substantial impact on vector reduction, reinforcing its role as a key preventive measure in visceral leishmaniasis control programs [32].

Numerous studies confirm that strategies such as canine vaccination and the use of insecticide-impregnated collars contribute meaningfully to VL control [8,33,34,35,36,37,38,39]. Strengthening these measures alongside community education can enhance program effectiveness and public awareness.

Successful VL control requires integrated interventions supported by interinstitutional partnerships, municipal capacity building, environmental and epidemiological surveillance, habitat modification, and behavior change communication. Among these, health education is pivotal in fostering community participation and sustainable disease prevention [29].

## 5. Conclusions

The results of this study highlight the urgent need to strengthen control strategies in the municipality of Araçatuba. Integrated efforts combining environmental management and educational initiatives can effectively contribute to reducing the density of sandfly vectors, thereby aiding in the control of VL. Furthermore, identifying priority areas for intervention enables more focused disease surveillance and monitoring, optimizing resource allocation and enhancing the impact of control measures.

This approach aligns with current public health recommendations that emphasize multifaceted control programs to mitigate VL transmission risks and improve outcomes for affected communities. Enhancing these coordinated activities is essential for achieving sustained reductions in VL incidence in endemic regions such as Araçatuba.

## Figures and Tables

**Table 1:** LV control activities carried out by the municipality from 2019 to 2021.

Area/LWA	Canine Samples			Env. Management		Number of Human Cases	Houses		
Number	Collected	Positive	(%)	Management Guidance	(%)		Visited Dwellings	Worked	(%)
1	150	14	9.3%	214	85.2%	2	432	251	58.1%
2	93	15	16.1%	135	90.6%	3	194	149	76.8%
3	1421	200	14.1%	2602	99.1%	2	4388	2625	59.8%
4	51	0	0%	174	89.6%	1	286	194	67.8%
5	1522	205	13.5%	3498	90.6%	4	8042	3,86	48.0%
6	128	12	9.4%	1277	98.3%	0	2494	1299	52.1%
7	43	3	7.0%	34	91.8%	1	69	37	53.6%
Total	3408	449	13.2%	7934	94.2%	13	15,905	8415	52.9%

Notes: 1. “Positive canine samples” refer to dogs seropositive for *Leishmania* (*L.*) *infantum*. 2. “Environmental Management Guidelines” refers to properties with conditions favorable to the development of sandflies, where residents received instructions on cleaning and organizing the environment. 3. “Worked houses”: properties where the agent carried out intra- and peridomestic actions. “Visited houses”: all properties included within the block where the activity was conducted.

**Table 2:** Estimates of RPcl for *Lu. longipalpis* per collection night using CDC in the municipality of Araçatuba, state of São Paulo, Brazil.

Variable	Coefficient	Standard error	RPcl	Confidence Interval	
Intercept	−1.8761 *	0.0291	0.1532	0.1447	0.1622
2007 to 2015			1		
2016 to 2018	−0.3635 *	0.0515	0.6952	0.6285	0.7690
2019 to 2021	0.3522 *	0.0782	1.4222	1.2202	1.6576
LWA5	−0.0567	0.1841	0.9449	0.6587	1.3554

* *p* < 0.05.

## Data Availability

The original contributions presented in this study are included in the article. Further inquiries can be directed to the corresponding author.
